# Identification of DreI as an Antiviral Factor Regulated by RLR Signaling Pathway

**DOI:** 10.1371/journal.pone.0032427

**Published:** 2012-03-07

**Authors:** Shun Li, Fan Sun, Yi-Bing Zhang, Jian-Fang Gui, Qi-Ya Zhang

**Affiliations:** State Key Laboratory of Freshwater Ecology and Biotechnology, Wuhan Center for Developmental Biology, Institute of Hydrobiology, Graduate School of the Chinese Academy of Sciences, Chinese Academy of Sciences, Wuhan, China; INRA, France

## Abstract

**Background:**

Retinoic acid-inducible gene I (RIG-I)–like receptors (RLRs) had been demonstrated to prime interferon (IFN) response against viral infection via the conserved RLR signaling in fish, and a novel fish-specific gene, the grass carp reovirus (GCRV)-induced gene 2 (*Gig2*), had been suggested to play important role in host antiviral response.

**Methodology/Principal Findings:**

In this study, we cloned and characterized zebrafish *Gig2* homolog (named *Danio rerio Gig2-I*, *DreI*), and revealed its antiviral role and expressional regulation signaling pathway. RT-PCR, Western blot and promoter activity assay indicate that *DreI* can be induced by poly I:C, spring viremia of carp virus (SVCV) and recombinant IFN (rIFN), showing that *DreI* is a typical ISG. Using the pivotal signaling molecules of RLR pathway, including RIG-I, MDA5 and IRF3 from crucian carp, it is found that *DreI* expression is regulated by RLR cascade and IRF3 plays an important role in this regulation. Furthermore, promoter mutation assay confirms that the IFN-stimulated regulatory elements (ISRE) in the 5′ flanking region of *DreI* is essential for its induction. Finally, overexpression of *DreI* leads to establish a strong antiviral state against SVCV and *Rana grylio* virus (RGV) infection in EPC (Epithelioma papulosum cyprinid) cells.

**Conclusions/Significance:**

These data indicate that DreI is an antiviral protein, which is regulated by RLR signaling pathway.

## Introduction

In mammals, recognition of viral components through pattern recognition receptors (PRRs) triggers several signaling cascades, eventually converging on the transcription activation of IFNs and ISGs against virus infection [1]. Three classes of PRRs have been identified: retinoic acid-inducible gene I (RIG-I)–like receptors (RLRs), Toll-like receptors (TLRs) and nucleotide oligomerization domain (NOD)-like receptors (NLRs). RLR family, the cytosolic sensors for viral RNA, comprises three helicases: RIG-I, melanoma differentiation-associated gene 5 (MDA5), and laboratory of genetics and physiology 2 (LGP2). Both RIG-I and MDA5 contain two N-terminal caspase recruitment domains (CARDs) to bind viral RNAs. However, LGP2, lacking two CARDs in the N-terminus, is identified as a negative regulator of RLR signaling pathway [2], [3]. RIG-I preferentially binds short double-stranded RNA (dsRNA) and 5′-triphosphate single-stranded RNA (ssRNA), whereas MDA5 only recognizes long molecules of dsRNA [4], [5]. Upon recognition of viral infection, RIG-I and MDA5 transmit the signals to downstream adaptor protein mitochondrial antiviral signaling protein (MAVS, also known as IPS-1/VISA/Cardif) [6], [7], [8], [9]. Subsequently, MAVS induces the activation of interferon regulatory factor 3/7 (IRF-3/7) and NF-κB, which together enter the nucleus to initiate the transcription of type I IFN genes and a subset of ISGs [10], [11].

Recently, several studies have suggested that the RLR signaling pathway also functionally exists in fish. Using tblastn search with *Homo sapiens* RLR protein sequences as bait, several fish RLR genes were mined in Ensembl database (http://www.ensembl.org). Zou et al presumed that RLR family might be conserved in vertebrates and the core function domains diversification might be essential to the function divergence of recognition of viruses [12]. A RIG-I-like molecule was identified in salmonid and cyprinid cell lines. Overexpression of RIG-I N terminal CARDs was able to establish a strong antiviral state against several DNA and RNA viruses by inducing the expression of ISGs [13]. MDA5 has been demonstrated to play virus recognition role in rainbow trout and Japanese flounder [14]. In crucian carp, overexpression either RIG-I or MDA5 was able to strongly upregulate fish virus-induced IFN production [15]. The third member of RLR family, LGP2, has been identified from rainbow trout and Japanese flounder, and its overexpression confers host cells powerful antiviral activity by induction of ISGs. However, crucian carp LGP2 was demonstrated to negatively regulate the RLR signaling pathway [14], [15], [16]. MAVS, downstream adaptor protein of RLRs, has also been cloned from several fish species including Japanese flounder, Atlantic salmon and zebrafish. Further experiments showed that the MAVS-overexpressed cells exhibited a severe inhibition of the replication of both DNA and RNA viruses [13], [17]. As mentioned above, the critical molecules of RLR signaling pathway in fish and mammals share the similar structure and function, however, several differences also exist. Fish mediator of IRF3 activation (MITA) is able to links the signal from RIG-I/MDA5 to downstream kinase TBKI and IFN transcription factor IRF3, but failed to induce the activation of NF-κB [15]. The transcription factor IRF3 is a critical protein downstream of RLR signal pathway, and regulates the expression of type I IFN and ISGs. A recent study shows that fish IRF3, unlike its mammalian orthologs, is a typical ISG which is significantly upregulated by rIFN, poly I:C, B-DNA and Z-DNA. Besides that, the phosphorylation and nuclear translocation of fish IRF3 were also observed in the condition of rIFN treatment, which did not occur in mammals [18]. Furthermore, grass carp reovirus (GCRV)-induced gene 2 (*Gig2*) has been identified as a novel fish-specific gene from the UV-inactivated GCRV-treated *Carassius auratus* blastulae embryonic (CAB) cells [19], and its transcription is upregulated by viral infection and IFN treatment. Subsequently, *Ca*Gig2 was presumed to play an important role in fish innate antiviral response because overexpression of IRF7 led to a strong activation of *Ca*Gig2 promoter [20]. These studies have revealed that *CaGig2* is an ISG that is regulated by IRF7, however, the subtle signal cascade for its induction and role in antiviral response are still unknown.

As a powerful vertebrate model for infectious disease and immune function, zebrafish has risen tremendous interests in fish immune response [21]. The homologues of *CaGig2* in zebrafish constitute a large gene family, according to the position order in chromosomes, they were named *Danio rerio Gig2*-*A* to *–Q* (*DreA* to *DreQ*), and *DreI* was the one with highest identity to *CaGig2*. In this study, we firstly mined *DreI* homolog from zebrafish genome database, and characterized its expression pattern, expressional regulation pathway and antiviral activity. Initially, the anti-DreI antiserum was generated and used to identify its expression profile and subcellular localization. Then, the 5′ flanking region of *DreI* was cloned in front of luciferase reporter gene to analyze its transcriptional activation by several stimuli including virus, poly I:C and rIFN, and some pivotal molecules of RLR signaling cascade, such as crucian carp *Ca*RIG-I, *Ca*MDA5 and *Ca*IRF3. Moreover, such activation could be severely impaired by the dominant negative mutant of CaIRF3 (designated *Ca*IRF3-DN). Furthermore, the ISRE motif found in *DreI* promoter was indispensable for the induction of *DreI* by several stimuli. Finally, the antiviral role of DreI in host immune response against both RNA and DNA viruses was demonstrated *in vitro*. These results provide a new insight into the antiviral response in lower vertebrate.

## Results

### Identification of zebrafish Gig2 homolog

**Figure 1:**
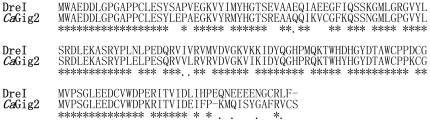
Alignment of amino acid sequences of DreI and *Ca*Gig2. The amino acid sequences of DreI and *Ca*Gig2 are deduced from the cDNA sequences (Accession numbers: *DreI*, HQ269376; *CaGig2*, No. GQ181131). The identical (*) and similar (.) amino acids identified by Clustal X program are indicated.

By searching zebrafish genome database with *CaGig2* sequence (accession No.GQ181131), several homologous *CaGig2* genes were found and the most identical one was named as *DreI* (accession No. HQ269376). Using PCR method, we obtained the full-length cDNA of *DreI* from a cDNA library of zebrafish liver (ZFL) cells stimulated with poly I:C. The open reading frame consists of 474 nucleotides (nt) and encodes 158 amino acids with approximately 76.1% identity to *Ca*Gig2. Additionally, neither nuclear localization signal nor transmembrane motif (TM) was found in DreI protein (Fig. 1).

### Inducible expression of *DreI* by poly I:C in vitro

**Figure 2:**
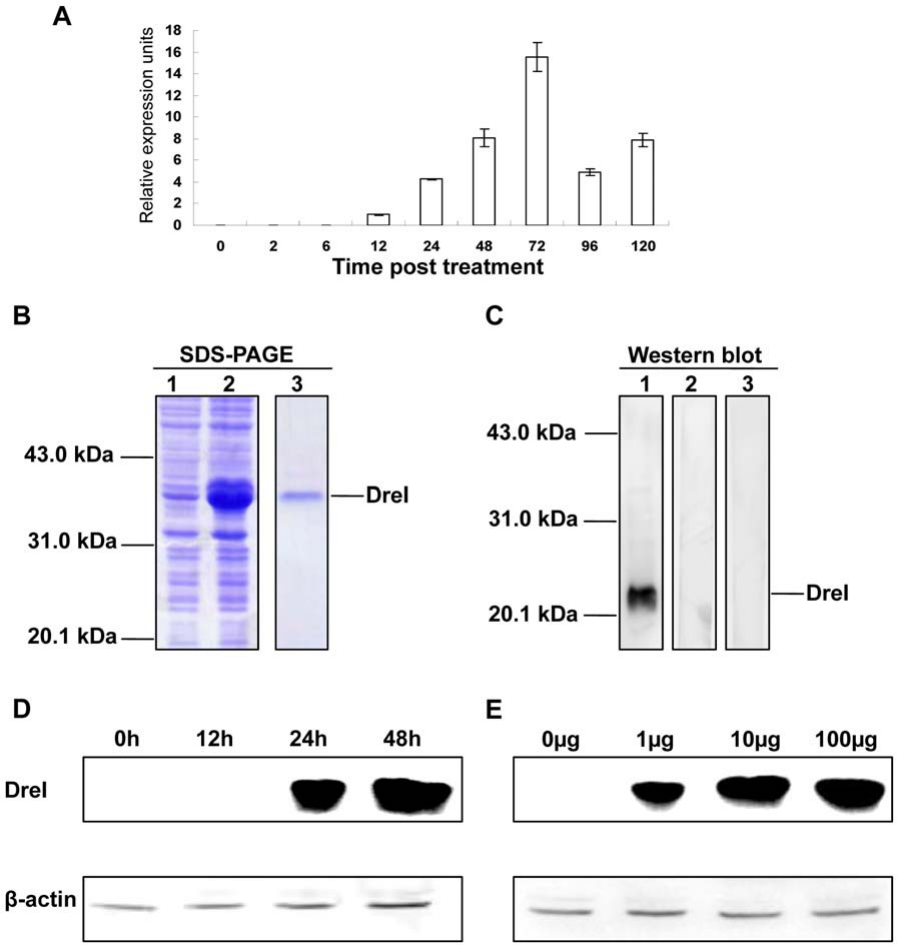
Inducible expression pattern of *DreI*. (A) ZFL cells seeded on 6-well plates overnight were transfected with 2 µg/ml poly I:C for 2, 6, 12, 48, 72, 96, and 120 h. Then total RNAs were extracted to examine the expression level of *DreI* transcripts by real-time PCR. *β-actin* was introduced as endogenous control. (B) Prokaryotic expression of the fusion protein DreI-His and generation of anti-DreI polyclonal antibody. Lane 1: lysate of normal bacteria; lane 2: lysate of IPTG-induced bacteria; lane 3: the purified protein by Ni^2+^-NTA affinity chromatography. (C) Transfection of ZFL cells with 2 µg/ml poly I:C for 48 h, the lysate was immunoblotted by polyclonal anti-DreI antiserum (lane 4), normal rabbit serum (lane 5) or anti-DreI antiserum pre-adsorbed with purified prokaryotic protein (lane 6). (D) ZFL cells were stimulated with 2 µg/ml poly I:C plus 4 µl/ml Lipofectamine 2000 for 12, 24, and 48 h, then lysed and detected by anti-DreI antiserum. β-actin served as an internal control. (E) For dose-dependent analysis, ZFL cells were treated with 1, 10, and 100 µg/ml poly I:C for 24 h. β-actin served as an internal control.

To investigate the expression pattern of *DreI*, real-time PCR was applied to detect its mRNA level. As shown in Fig. 2A, transient transfection with poly I:C was able to induce the transcription of *DreI* in ZFL cells at 12 h, and to reach the peak at about 72 h. To further clarify the expression of DreI at protein level, the full-length ORF of *DreI* was expressed in *E. coli* as a His tagged fusion protein with about 39 kD (Fig. 2B, lane 2), and the purified DreI-His protein (Fig. 2B, lane 3) was used to immunized white rabbit to generate a polyclonal anti-DreI antiserum. For specificity detection, the poly I:C treated ZFL cell lysate was analyzed with this antiserum by Western blotting. As shown in Fig. 2C, the anti-DreI antiserum from the immunized rabbit rather than the serum from the pre-immunized rabbit could detect the endogenous DreI protein with a molecular weight of appropriate 22 kDa which is different with that of the fusion protein (about 39 kDa). Moreover, when the anti-DreI antiserum was preabsorbed with the fusion protein DreI-His, it could not recognize the 22 kDa protein band. Therefore, the produced antiserum was subsequently used to monitor the expression of DreI protein. In accordance with the induction kinetics at mRNA level, DreI protein was also significantly induced by transfection of poly I:C in ZFL cells since 24 h (Fig. 2D). Furthermore, ZFL cells were treated with various concentrations of poly I:C from 1 µg/ml to 100 µg/ml for 24 hours. As shown in Fig. 2E, the DreI protein was greatly upregulated by polyI:C treatment in a dose-dependent manner. For the limitation on the highly related protein family recognition of polyclonal antiserum, other member(s) besides DreI might contain in the band. These results indicate that *DreI* can be induced by poly I:C both at mRNA and protein levels.

### Analysis of *DreI* promoter

**Figure 3:**
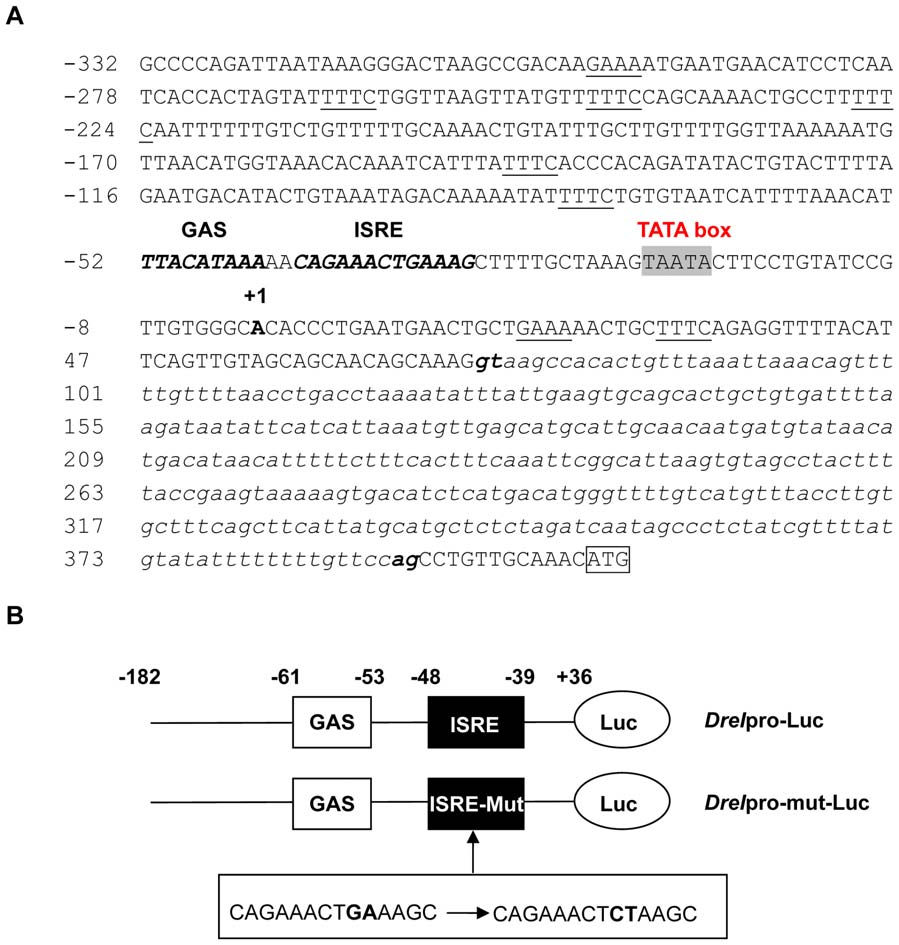
Analysis of the 5′ flanking regulatory sequence of *DreI*. (A) The 5′ flanking region of *DreI* was mined from NCBI public database. The putative transcription start site was in bold and the start code of the *DreI* transcript was boxed. The intron in 5′UTR of *DreI* is indicated with lower case italics and the conserved flanking GT/AG dinucleotides are shown in bold. The putative ISRE motif and GAS site were in italic type. The TATA box is shaded in grey and Eight GAAA/TTTC motifs are underlined. (B) The schematic of 5′ flanking region of *DreI*. To analyze *DreI* promoter activity, the region containing ISRE and GAS motifs was cloned in front of luciferase reporter gene. To construct the ISRE mutant promoter, the purine G and A in −59 and −58 were mutated to pyrimidine C and T, respectively.

To further characterize the expression of *DreI*, we searched 5′ flanking region about 700 bp upstream of the start codon of *DreI* in zebrafish genome database. As shown in Fig. 3A, the putative transcription start site (A) is localized at 388 bp upstream from the translation start codon of *DreI* and determined as position +1. An intron of 319 bp (+71 to +390) was found to exist in the 5′UTR, which contains the conserved flanking dinucleotides GT-AG in the exon/intron structure. Moreover, promoter sequence characterization analysis revealed several putative transcription factor binding sites in the 5′ flanking regulatory region. A putative ISRE motif, present in the promoters of most ISGs, was identified in the region from −48 to −39, which matches the consensus sequence of (G/A/T)GAAAN(1–2)GAAA(G/C)(A/T/C) [22]. A gamma IFN activated sequence (GAS) site (TTNCNNNAA) and eight GAAA/TTTC motifs were also found in the *DreI* promoter, which are the characteristics of genes responsive to both type I IFN and type II IFN (Fig. 3A).

**Figure 4:**
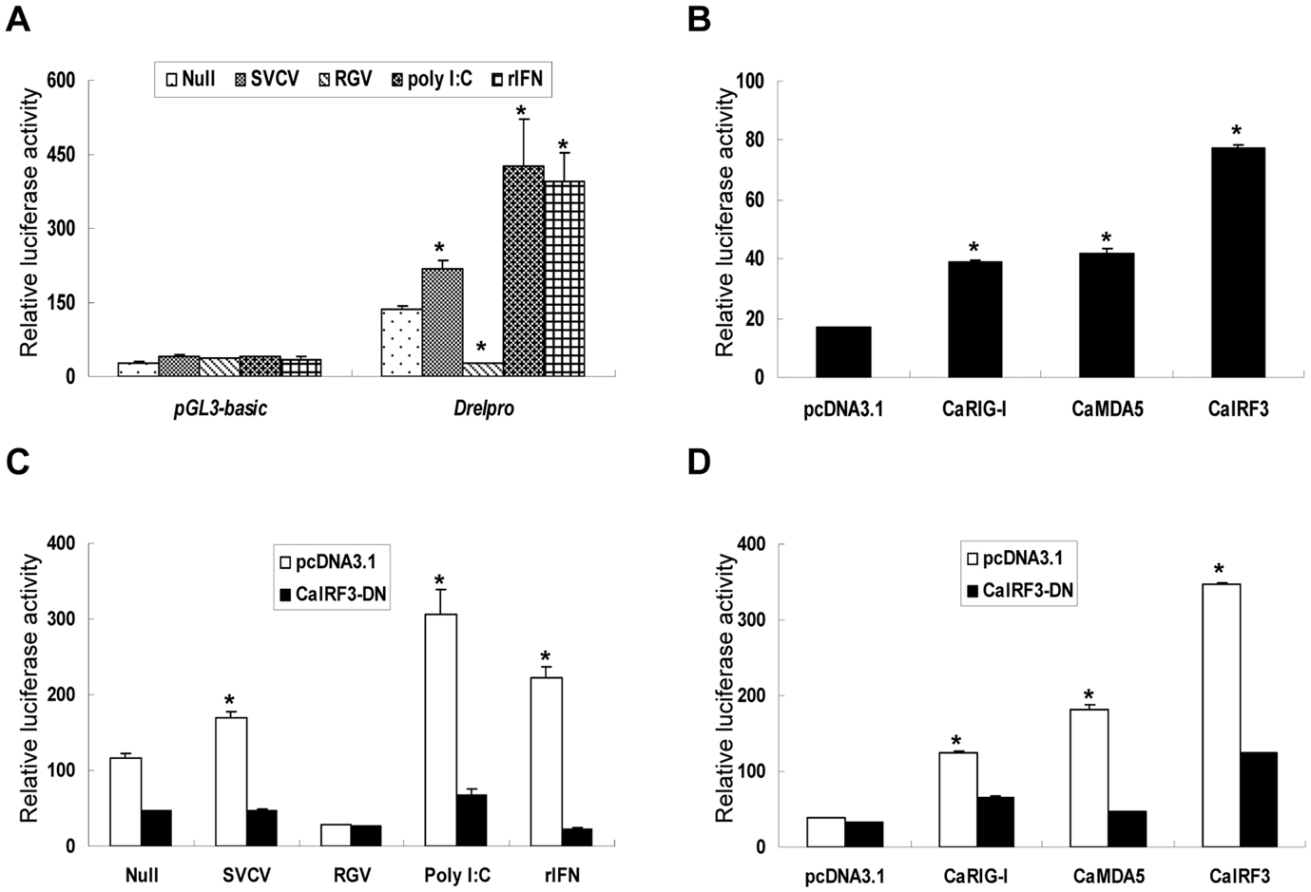
Activation of *DreI* promoter by several stimuli via RLR signaling. (A) Induction of *DreI* promoter by SVCV, RGV, rIFN and poly I:C for 24 h. EPC cells seeded in 24-well plates overnight were transfected with *DreI*pro-Luc, and pRL-TK was used as an internal control. At 24 h after transfection, cells were treated with several stimuli or left untreated. The luciferase activities were monitored at 24 h after stimulation. (B) EPC cells were cotransfected with pcDNA-*CaRIG-I*, pcDNA-*CaMDA5*, or pcDNA-*CaIRF3* and *DreI*pro-Luc at the ratio of 1∶1. The luciferase activity was assessed at 48 h post-transfection. (C) EPC cells were cotransfected with *DreI*pro-Luc and pcDNA-*CaIRF3-DN* and stimulated with several stimuli at 24 h posttansfection. Luciferase activities were monitored at 24 h after stimulation. (D) EPC cells were cotransfected with pcDNA-*CaRIG-I*, pcDNA-*CaMDA5*, or pcDNA-*CaIRF3* and *DreI*pro-Luc plus pcDNA-*IRF3-DN* at the ratio of 1∶1∶1. At 48 h posttransfection, cells were collected for detection of luciferase activates. The results represent three independent experiments and error bars are the SDs obtained by measuring each sample in triplicate. The significant differences between control and treatments groups are determined by T-TEST. *p<0.01.

To analyze the promoter activity, about 200 bp minimal promoter sequence from −182 to +36 containing the putative GAS site and ISRE motif was cloned in front of luciferase reporter gene to generate the *DreI* promoter-driven luciferase construct, which was designated *DreI*pro-Luc (Fig. 3B). In comparison with pGL3-basic vector, the relative luciferase activities were greatly elevated in the *DreI*pro-Luc transfected EPC cells, indicating that the cloned *DreI* promoter is a strong promoter. (Fig. 4A). As anticipated, when the *DreI*pro-Luc transfected EPC cells were treated or infected by poly I:C, rIFN, or SVCV, the relative luciferase activities were further induced. As shown in Fig. 4A, compared to the untreated (null) cells, about 3.07-fold, 2.86-fold and 1.58-fold activation of *DreI* promoter were induced in the presence of poly I:C transfection, rIFN treatment and SVCV infection, respectively. However, none inducible activities were observed in the transfected cells with control pGL3-basic vector. Intriguing, RGV infection did not induce the activation of *DreI*pro-Luc, and even inhibited its basal luciferase activity. It was likely that RGV had evolved some mechanisms to avoid or impede host immune response. These results together indicate that *DreI* promoter was significantly activated by poly I:C, rIFN and SVCV.

### Regulation of *DreI* expression via RLR signaling

Recently, great progress has been made in characterization of fish RLR signaling pathway response to virus infection. In crucian carp, a functional RLR-activated signaling cascade has been found to be essential for IFN response against viral infection [20]. To determine whether *DreI* was regulated through RLR signaling pathway, *CaRIG-1*, *CaMDA5* and *CaIRF3* were employed in the current study. As shown in Fig. 4B, overexpression of *CaRIG-I* and *CaMDA5* led to a significant activation of *DreI* promoter (2.31 and 2.47 fold relative to the empty vector respectively). Moreover, a 4.62-fold luciferase activity of *DreI*pro-Luc was induced by overexpression of *CaIRF3* in comparison with that of control vector. These data indicate that the expression of *DreI* was upregulated by *CaRIG-I*, *CaMDA5* and *CaIRF3*.

In a previous study, fish IRF3 has been demonstrated as a crucial transcription factor for IFN and ISG expression downstream of RIG-I and MDA5 [15]. To delineate the role of CaIRF3 in the regulation of *DreI* expression, *CaIRF3-DN*, a dominant negative form of *CaIRF3*, was used in this study. As shown in Fig. 4C, the induction of *DreI* promoter by SVCV, poly I:C and rIFN was severely impaired by overexpression of *CaIRF3-DN*, indicating that CaIRF3 was indispensable for *DreI* induction by such mentioned stimuli. Furthermore, the activation of *DreI* promoter induced by *CaRIG-I*, *CaMDA5* and *CaIRF3* (3.28, 4.76 and 9.13 fold respectively) was also significantly inhibited by overexpression of *CaIRF3-DN*, resulting in 1.43-, 2.01- and 3.89-fold reduction, respectively (Fig. 4D). These results together indicated that RLR-activated signaling cascade was essential for *DreI* expression upon viral infection and IRF3 was probably the master transcription factor for the inducible transcription of *DreI*.

### ISRE motif is indispensable for *DreI* induction

**Figure 5:**
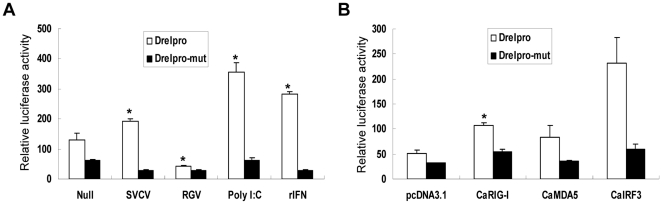
ISRE is essential for the induction of *DreI* promoter. (A) EPC cells seeded in 24-well plates overnight were transfected with *DreI*pro-Luc or *DreI*pro-mut-Luc plus pRL-TK and induced by SVCV, RGV, rIFN and poly I:C for 24 h. Cells were collected for measuring the luciferase activities. (B) EPC cells were cotransfected with pcDNA-*CaRIG-I*, pcDNA-*CaMDA5*, or pcDNA-*CaIRF3* and *DreI*pro-mut-Luc at the ratio of 1∶1. In the control group, the EPC cells were cotransfected with indicated expression constructs and *DreI*pro-Luc at the ratio of 1∶1. At 48 h posttransfection, the cells were collected to assess the luciferase activates. The results are the representative of three independent experiments and error bars are the SDs obtained by measuring each sample in triplicate. *p<0.01.

Since ISRE motif in promoter of ISGs is essential for its transcriptional activation upon stimulation, an ISRE mutant *DreI* promoter-driven luciferase construct (designated as *DreI*pro-mut-Luc) was made to clarify whether the ISRE motif was necessary for *DreI* induction. EPC cells were transfected with wild type or ISRE mutant *DreI* promoter construct followed by treatment with poly I:C or rIFN and by infection with SVCV or RGV. Similar to previous experiments, wild type *DreI* promoter was significantly activated by poly I:C, rIFN, and SVCV, whereas no significant activation of *DreI*pro-mut-Luc was observed in the same condition (Fig. 5A). These results suggested that the ISRE motif was indispensable for *DreI* transcription activation induced by several stimuli.

Furthermore, we also determined the role of ISRE motif in the activation of *DreI* promoter triggered by *CaRIG-I*, *CaMDA5* or *CaIRF3*. As shown in Fig. 5B, consistent with previous results, overexpression of *CaRIG-I*, *CaMDA5*, and *CaIRF3* led to a remarkable activation of *DreI* promoter (2.14, 1.66, and 4.62 fold, respectively), but such activation was severely inhibited when it came to *DreI*pro-mut-Luc (1.63, 1.21, and 1.68 fold, respectively). Collectively, these results demonstrated that the ISRE motif was the major cis-element indispensable for *DreI* induction.

### Antiviral activity of *DreI* protein

**Figure 6:**
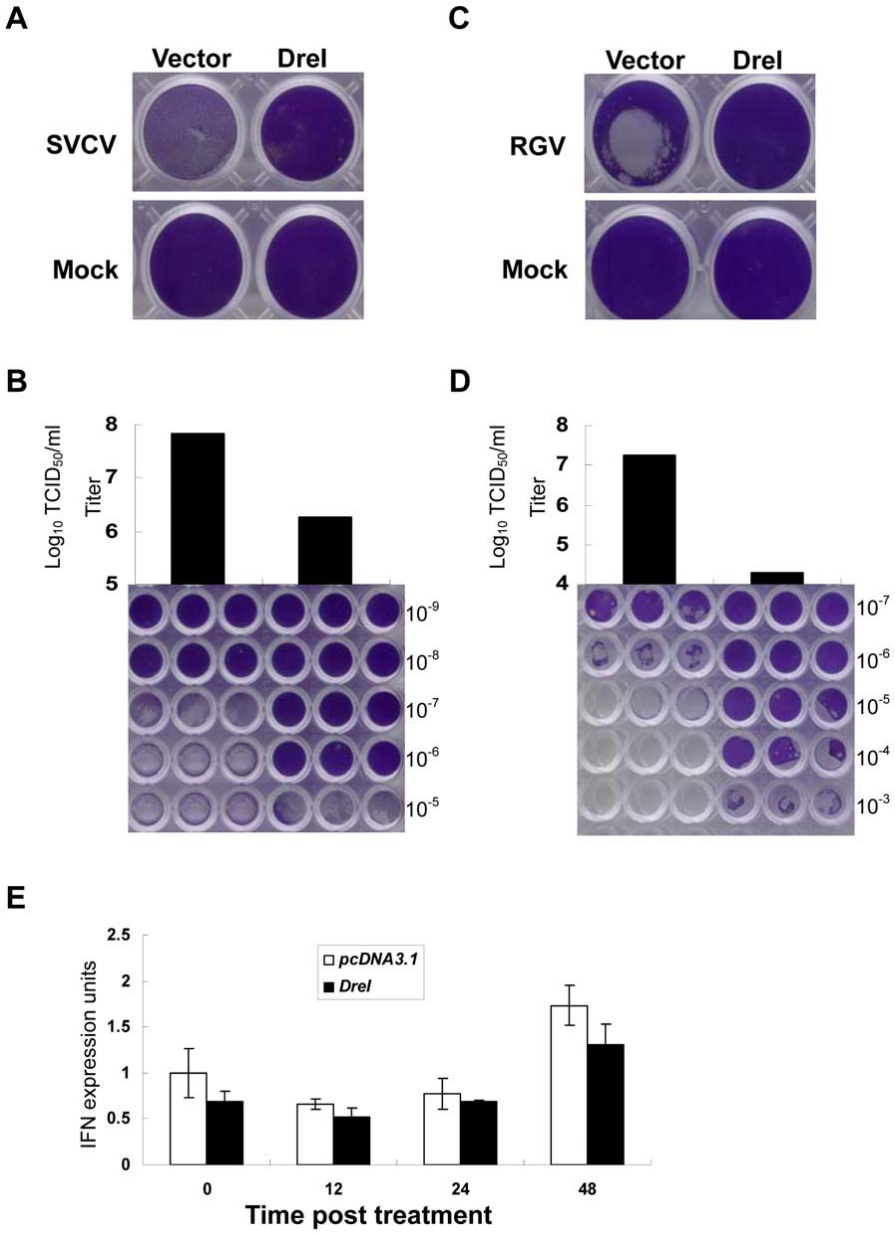
Overexpression of *DreI* induces powerful antiviral immunity. (A, C) EPC cells seeded in 24-well plates overnight were transfcted with 0.5 µg *DreI* plasmid or empty vector as a control. At 24 h postransfection, EPC cells were infected with SVCV or RGV at a dose of 10 TCID50 per well for 5 days at 25°C. Then Cell monolayers were stained with crystal violet. (B, D) The culture supernatants from cells infected with SVCV and RGV were collected and the viral titers were measured by standard TCID_50_ method. The CPE caused by SVCV or RGV in viral titer measurement assays was also presented. The results are the representative of two independent experiments. (E) EPC cells were transfected with empty vector or *DreI* plasmid and were sampled at the indicated times. The relative transcript level of IFN was detected by real-time PCR and normalized to the expression of β-actin. Error bars represent SDs obtained by measuring each sample in triplicate.

Since *DreI* is indeed a typical ISG, the role of DreI protein should be determined in cellular antiviral response against viral infection. EPC cells were transfected with *DreI* or empty vector as a control followed by infection with SVCV or RGV, which belong to RNA or DNA virus, respectively. At five-day postinfection with SVCV, an obvious broad CPE was observed in control cells, whereas overexpression of *DreI* fully protected cells against SVCV infection (Fig. 6A). Consistently, the SVCV virus titer was remarkable decreased about 100-fold (10^6^ TCID_50_/ml versus 10^8^ TCID_50_/ml) in the *DreI*-transfected cells compared to that of control cells (Fig. 6B). On the other hand, overexpression of *DreI* was able to greatly delay the appearance of CPE resulted from RGV infection (Fig. 6C). Moreover, a viral titer of 4.2×10^4^ TCID_50_/ml was detected in the supernatant from the *DreI*-overexpressed cells, which was a 1740-fold reduction compared to that of control cells (7.3×10^7^ TCID_50_/ml; Fig. 6D). Finally, a real-time PCR was performed to detect the induction of IFN in EPC cells by overexpression of *DreI*. As shown in Fig. 6E, *DreI* indeed functional as a final effector. Therefore, the data indicate that DreI is an antiviral protein against both RNA and DNA viruses in fish cells.

### DreI is localized in the cytoplasm

**Figure 7:**
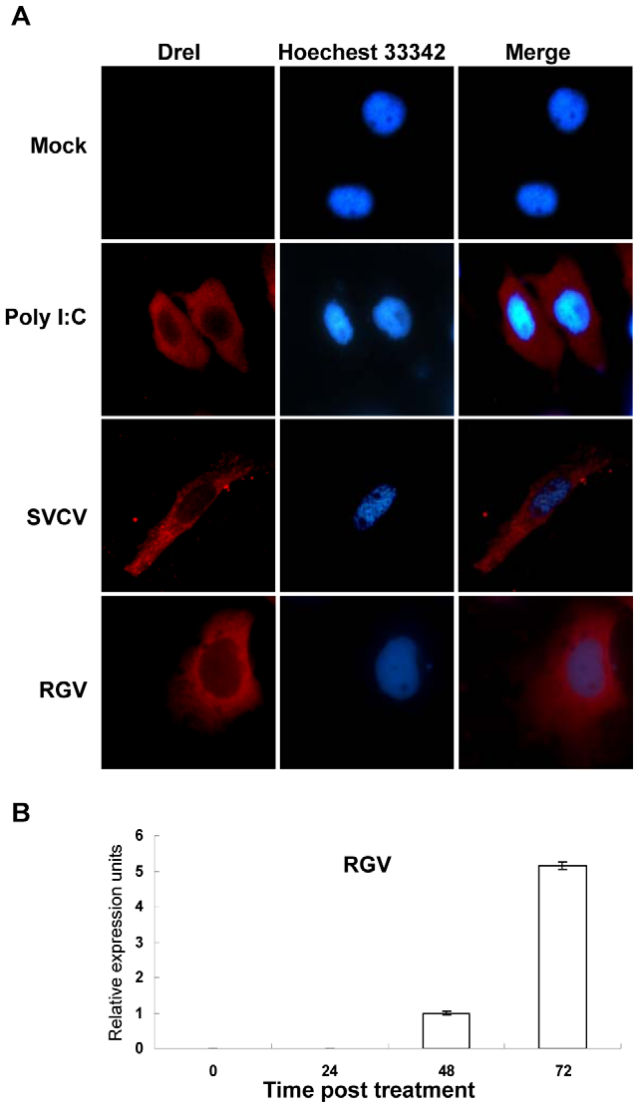
Immunofluorescence localization of DreI. (A) ZFL cells seeded on microscope cover glass in 6-well plates were stimulated with 2 µg poly I:C for 24 h, or infected with 10 TCID_50_ of RGV or SVCV per well for 72 h, then the cells were fixed, permeabilized, and immunoblotted with anti-DreI antiserum at 4°C overnight. The cell monolayers were further stained with Alexa Fluor 546 anti-rabbit antibody and Hoechest 33342, then examined using a Leica DM IRB fluorescence microscope. Mock-infected ZFL cells were used as a control. The red staining represented DreI protein signal and the blue indicated nucleus region. Magnification 100 (oilimmersion objective). (B) ZFL cells were infected with RGV at a dose of 10 TCID_50_ and were sampled at the indicated times. The relative transcript level of *DreI* was detected by real-time PCR and normalized to the expression of *β-actin*. Error bars represent SDs obtained by measuring each sample in triplicates.

To determine the cellular localization of DreI, immunofluorescent microscopy was used to examine the localization of DreI in ZFL cells. Since DreI protein did not express in normal ZFL cells as described above, ZFL cells plated on microscopic glass overnight were stimulated with poly I:C, SVCV, RGV or not. At 72 h poststimulation, the DreI protein was detected by polyclonal anti-DreI rabbit serum. As anticipated, no signal was monitored in the untreated ZFL cells, however, the red signals of DreI were markedly observed to be widespread in the cytoplasm when the cells were stimulated with poly I:C, or infected with SVCV or RGV (Fig. 7A). Because of the discrepancy between positive staining of DreI protein and negative effect of *DreI* promoter in the context of RGV infection, a real-time PCR was performed to monitor the expression of *DreI* mRNA by RGV infection. As shown in Fig. 7B, *DreI* transcription is intensively upregulated from 48 h after RGV infection. These results indicate that DreI is a cytoplasmic protein.

## Discussion

In the past two decades, an immense progress has been made in identification of IFNs and ISGs in several fish species, and lots of findings indicate that fish, similar to mammals, also possesses a functional conserved IFN system to inhibit viral infection [21]. Although a great number of ISGs had been identified in response to IFN from several fish species, only a few of them have been characterized in the expression regulation and antiviral function. Herein, we have identified DreI as a typical ISG protein, which is regulated by the conserved RIG-I/MDA5 signaling through transcription factor IRF3, and demonstrated its antiviral activity against both DNA and RNA viruses. This is the first study to determine the expression regulation and antiviral function of *DreI*.

Fish type I IFN system exerts a pivotal role in host defense against virus infection, and the induction of IFNs and ISGs is considered as the hallmark of antiviral innate immune response [23]. Upon viral infection, the cytosolic receptors RIG-I and MDA5 can detect viral genome RNA or replication byproduct dsRNA to induce multiple signaling pathways, and eventually activate transcription factors IRF3 and NF-κB, which subsequently translocate into nucleus and initiate IFN transcription. Through an autocrine/paracrine loop, a large number of ISGs are activated by IFN [24]. In the present study, the expression of *DreI* was shown to be significantly unregulated by poly I:C at mRNA level. Furthermore, a specific polyclonal antibody against DreI was generated and used to confirm the expression profile of DreI protein. Western blotting analysis showed that DreI protein was induced by poly I:C treatment in both time- and dose-dependent manners. It is worthy of note that no signal of *DreI* mRNA or protein was detected in untreated ZFL cells. Therefore, *DreI* seems to be an ISG gene induced by poly I:C and IFN. On the other hand, characterization of 5′ flanking regulatory sequence of *DreI* reveals multiple transcription factor binding sites including ISRE and GAS. Luciferase reporter assay exhibits that the activity of *DreI* promoter is remarkably upregulated by poly I:C, rIFN and SVCV infection. Collectively, these results together indicate that *DreI* is a typical ISG gene. Recently, the cytosolic viral RNA sensors RIG-I/MDA5-mediated signaling pathway responsible for type I IFN activation was functionally demonstrated in some fish species. Some pivotal signaling molecules, including RIG-I, MDA5, MITA, TBK1 and IRF3, have been cloned and characterized in crucian carp [15]. To elucidate subtle signaling pathway responsible for *DreI* induction, several crucian carp molecules, such as RIG-I, MDA5 and IRF3, were used in the current study. In reporter assays, a strong activation of *DreI* promoter was observed when the cells were transfected with any molecule among *Ca*RIG-I, *Ca*MDA5 and *Ca*IRF3 even in the absence of poly I:C or rIFN stimulation. *Ca*IRF3 is an important transcription factor for the induction of IFN and ISGs, whereas *Ca*IRF3-DN, devoid of DNA binding domain, exhibits a dominant negative effect. This is probably due to its ability to interact with endogenous TBK1, IRF7 or itself, and thus abolishes the function of endogenous IRF3. As anticipated, the activation of *DreI* promoter induced by overexpression of *Ca*RIG-I, *Ca*MDA5 and *Ca*IRF3 was severely abrogated by *Ca*IRF3-DN overexpression. It means that the expression of *DreI* is IRF3-dependent and is regulated by RLR pathway. In a previous study, *Ca*IRF7 was also demonstrated as a strong transcription factor for *Ca*Gig2 expression [20]. Taken together, these date verify the role of the conserved RLR signaling cascade in the activation of IRF3/7-dependent IFN and ISGs. Although the expression of ISGs activated by virus-induced IFN through the conserved Jak-Stat signaling cascade was also confirmed in fish [23], the further study is needed to confirm that the Jak-Stat pathway also is also involved in *Ca*Gig2 induction.

Previous studies have shown that more than 300 ISGs like *DreI* have been identified in response to IFN. However, only a few of them have been characterized for the function to resistant to virus invasion by affecting virus transcription, replication or cell apoptosis. For example, Mx, an IFN-inducible large GTPase belonging to the dynamin family, can inhibit various viruses by blockading capsid protein transposition and viral RNA synthesis [25], [26]. PKR encodes a protein that contains a dsRNA binding region and a protein translation inhibition region. Overexpression of *Po*PKR increases phosphorylation of the alpha subunit of eukaryotic initiation factor 2 (eIF2α) to inhibit viral protein synthesis [27], [28]. Viperin, an IFN-inducible protein, exerts its antiviral effect by inhibiting human cytomegalovirus (HCMV) maturation and assembly [29]. SVCV and RGV distribute worldwide and belong to RNA and DNA aquatic viruses respectively, and both of them could induce cell apoptosis *in vitro* and lead to high mortality in the infected fish [30], [31]. In the current study, overexpression of *DreI* conferred host cells to establish a strong antiviral state to inhibit the replication of both RNA and DNA viruses. Although *DreI* displays a powerful antiviral activity against viral infection, its underlying mechanisms are still elusive. Since subcellular localization plays an important role, we subsequently detected the distribution of DreI *in vitro* under the condition of viral infection. In line with results of *DreI* induction at mRNA and protein levels, immunoflurescence localization revealed cytoplasm distribution of DreI upon viral infection, and no signal was observed in normal cells, although its exact localization, such as mitochondrium or endoplasm reticulum, remained unknown. Collectively, it would be concluded that DreI inhibits viral infection in cytoplasm, and future study is dedicated to identify the viral protein(s) interacted with DreI.

Recently, the major components of type I IFN system, ISGs and its regulatory element ISRE, were identified to be conserved in teleost fish. The ISRE motif was found to be crucial for the induction of most fish ISGs, such as *Mx*. Deletion mutational analysis suggested that the ISRE motif closest to the transcription start site might be necessary for promoter activity of *Mx* gene [22], [32], [33], [34]. Similar situations were also found in the ISRE motif within promoters of Japanese flounder *ISG15* and crucian carp *IFN* [17], [35]. Consistent with other ISGs promoter, the 5′ flanking region of *DreI* also contains a typical ISRE motif. Mutation assays showed that the ISRE motif disruption completely abolished the activation of *DreI* promoter induced by SVCV, poly I:C and rIFN, confirming its essential role within *DreI* promoter. Furthermore, a high level of *DreI* promoter activity was monitored even without poly I:C transfection in luciferase assay, but no any signal of *DreI* mRNA or protein was detected in absence of poly I:C stimulation. These results indicate that *DreI* promoter is a strong promoter and seems to lack the negative regulatory elements. Such situation of high basal expression level also occurred in *CaGig2* promoter and zebrafish IFN3 promoter [15], [20]. Interesting, in luciferase assay, *DreI* promoter activity was not activated, but rather inhibited severely by RGV infection at 24 h postinfection. However, immunofluorescent localization experiments showed that the expression of DreI protein was significantly induced by RGV infection at 72 h postinfection. There are three explanations for such conflictive phenomena mentioned above. First, compared with ssRNA virus SVCV, a delayed appearance of host innate antiviral IFN response was probably induced by dsDNA virus RGV. Since poly dAT:dAT (B-DNA) and poly dGC:dGC (Z-DNA) induced the expression of IFN and ISG with slower kinetics than poly I:C [18]. Secondly, the *DreI* promoter we cloned was not long enough, and seemed to contain additional positive regulatory elements, such as IRF- or NF-kB-binding sites. The activity of *DreI* promoter containing only one ISRE motif could be easily inhibited by viral proteins synthesized by RGV. Finally, it was likely that the antiviral IFN response was blocked by RGV in the early stage of infection, but activated by subsequently synthesized viral products in the late stage. There are a lot of examples that, during the infection, many viruses synthesize some viral proteins to antagonize the innate immune response. Paramyxoviruses V protein was demonstrated to combine with cytosolic RNA sensor MDA5 to inhibit viral recognition and virus-triggered type I IFN signaling pathway [36]. NS3/4A encoded by hepatitis C virus was revealed to degrade the adaptor protein Cardif to block the RIG-I/MDA5-activated antiviral response [37], [38], [39].

Most of the ISGs identified in fish are conserved in vertebrates, but some are restricted to vertebrate subsets. *CaGig2*, a gene identified from subtractive suppression hybridization cDNA library stimulated with UV-inactivated GCRV, is a good case of such ISG restricted to some primitive vertebrates. By searching the EST and genome databases available at NCBI and Ensembl, several predicted genes homologous to *DreI* were found only in some aquatic vertebrate species, such as *Tetraodon nigroviridis* (accession No., CR719319), *Sebastes schlegelii* (accession No., AB490858), and *Xenopus* (accession No., XM_002932940). Considering the antiviral activity of DreI, primitive vertebrates might possess specific mechanism(s) to inhibit viral infection, which is probably lost in higher vertebrates during immune system evolution. It is interesting to clarify why *Gig2* gene is absent in reptiles, birds and mammals and whether the absence reduces the chance of aquatic virus infection when they transit their habitat from water to land [40]. Therefore, Gig2 may be an antiviral protein specific to aquatic viruses, and may have been lost during the evolution.It is plausible that DreI is an ancestral gene, which would not be retained in modern species of higher vertebrate branches. The migration of ancient tetrapods from water to land is likely a transition point for the disappearance of *DreI*.

Taken together, our study have characterized *DreI* as an antiviral ISG which is regulated by the conserved RIG-I/MDA5 signaling pathway through transcription factor IRF3, and demonstrated that the ISRE motif is indispensable for its induction. We also illustrate the essential role of DreI in host innate immune response against both RNA and DNA viruses. Further studies should be required to specify the interaction between DreI and viral proteins to illuminate its antiviral mechanisms.

## Materials and Methods

### Cells viruses, and rIFN

Epithelioma papulosum cyprinid (EPC) cells were cultured at 25°C in medium 199 supplemented with 10% fetal calf serum (FCS) [41]. ZFL cells were purchased from the American type culture collection (ATCC) and grown at 28°C in Leibovitz's L-15 medium with 2 mM L-glutamine, 50%; Dulbecco's modified Eagle's medium with 4.5 g/L glucose and 4 mM L-glutamine, 35%; Ham's F12 with 1 mM L-glutamine, 15% and supplemented with: 0.15 g/L sodium bicarbonate, 15 mM HEPES, 50 ng/ml EGF, heat-inactivated fetal bovine serum, 5%.

Spring viremia of carp virus (SVCV) [42], a negative ssRNA virus, and *Rana grylio* virus (RGV), a DNA virus, were maintained in our lab and propagated in EPC cells [43]. For viral infection, EPC cells seeded in 24-well plates overnight were transfected with 0.5 µg *DreI* or empty vector. At 24 h posttransfection, the cells were infected with 10 TCID_50_ of SVCV or RGV per well. Five days later, the supernatant aliquots were harvested for measurement of virus titers by the strandard TCID_50_ method. And then the cell monolayers were washed with PBS, fixed by 30% formaldehyde for 30 min, stained by 1% (w/v) crystal violet for 30 min, and observed for cytopathic effect (CPE). The results were the representative of three independent experiments. The source of rIFN was described previously [23].

### Gene cloning and plasmids

The open reading frame (ORF) of *DreI* was amplified using two pairs of primers by PCR from ZFL cells stimulated with poly I:C and cloned into the Kpn I and Xho I sites of pcDNA3.1/*myc*-His(−)A vector (Invitrogen) for overexpression, and into pET-32a(+) vector (Novagen) for prokaryotic expression, respectively. For promoter activity analysis, genome DNA was extracted from the zebrafish tail fin by Wizard® Genomic DNA Purification Kit (Promega). The 5′ flanking regulatory region (−200 to +24) of *DreI* was cloned and inserted into *pGL3-Basic* luciferase reporter vector (Promega). For mutation assay, the purine G/A (−59/−58) in the putative ISRE motif were mutated to pyrimidine C/T, respectively. *Ca*RIG-I, *Ca*MDA5, *Ca*IRF3 and *Ca*IRF3-DN were previously described [15]. All constructs were verified by sequencing analysis. The used primers are listed in Table 1.

**Table 1:** Primers used in this study.

Primers	Sequence (5′-3′)	Application
EE-DreI-F	GGTACCAACATGTGGGCTGAAGATGACT	overexpression
EE-DreI-R	GAATTCTCAGAAAAGTCTGCAACCATTT	
PE-DreI-F	GAATTCATGTGGGCTGAAGATGAC	Prokaryotic expression
PE-DreI-R	CTCGAGGAAAAGTCTGCAACCATTT	
DreI-Pro-F	TGGGTACCTTGGTTAAAAAATGTTAACA	Promoter activity analysis
DreI-Pro-R	AACTCGAGTGAAAGCAGTTTTTCAGCA	
DreI-mutPro-F	CATAAAAACACTAACTGAAA	mutation assay
DreI-mutPro-R	TTTCAGTTAGTGTTTTTATG	
DreI-F	GAAGACCAGAGAGTGATTGT	Real-time PCR
DreI-R	AACCATTTTCCTCTTCTTCG	
IFN-F	ATGAAAACTCAAATGTGGACGTA	
IFN-R	GATAGTTTCCACCCATTTCCTTAA	
β-Actin-F	CACTGTGCCCATCTACGAG	Real-time PCR, control
β-Actin-R	CCATCTCCTGCTCGAAGTC	

### RNA extraction and real-time PCR

Total RNA was extracted from ZFL cells by TRIZOL Reagent (Invitrogen). The first-strand cDNA was synthesized using random primers and M-MLV reverse system (Promega). The semi-quantitative RT-PCR was performed in a volume of 20 µl containing 1 µl cDNA, 0.2 µM each primer, 0.5 U of Taq polymerase (MBI, Ferments), 0.1 µM of dNTP and 1× buffer for Taq polymerase (MBI, Ferments). PCR was conducted with the protocol: 94°C for 4 min; then 94°C for 30 s, 54°C for 30 s, and 72°C for 30 s for 25–28 cycles; 72°C for 5 min. β-actin was introduced as an endogenous control gene. Real-time PCR was performed on ABI Step One real-time PCR system (Applied Biosystems, Foster City, CA, USA) with a dsDNA-binding dye, Fast SYBR green master mix (Applied Biosystems, Foster City, CA), All amplifications were performed using a two step temperature profile with annealing and extension at 60°C. The expression levels of mRNA were normalized by the median expression of β-actin. Each sample was analyzed in triplicates.

### Identification of the putative transcription start site

The putative transcription start site of *DreI* were identified by several softwares, including GENSCAN Web Server at MIT (http://genes.mit.edu/GENSCAN.html), Promoter 2.0 Predication Server (http://www.cbs.dtu.dk/services/Promoter/), Neural Network Promoter Predication (http://fruitfly.org:9005/seq_tools/promoter.html), and Promoter Scan (http://thr.cit.nih.gov/molbio/proscan/), and no any putative transcription start site was found between the ISRE motif and the first intron. It was likely that *DreI* has a noncanonical promoter such that was unable to identify by current softwares. However, we have retrieved several EST sequences (like HQ269376, EH281212, EH278211) from NCBI database, and found that one EST sequence (HQ269376) is transcribed from the Adenosine (A) about 250 bp upstream of translation start code. Moreover, a noncanonical TATA box (TAATA) probably recognized by RNA polymerase II was observed to locate at 26 nt ahead of the mentioned Adenosine (A). Taken together, we presumed that the Adenosine (A) might be the transcription start site and designated it as position +1.

### Transfection and luciferase reporter assays

EPC cells seeded in 24-well plates overnight were cotransfected with the mixture containing 0.525 µg plasmid and 1 µl FuGENE® HD Transfection Reagent (Roche) in 50 µl OPTI-MEM® I Reduced Serum Medium (Invitrogen). At 6 h after the poly I:C transfection or other stimulation, the cells were washed three times and cell medium was replaced by fresh 10%-FCS199 medium. The amount of expression vector, *DreI*pro-Luc and pRL-TK was at a ration of 10∶10∶1. pRL-TK vector was used to normalize the expression level. At 48 h posttransfection, the cells were harvested and lysed according to the Dual-Luciferase Reporter Assay System (Promega). Luciferase activities were measured by Junior LB9509 Luminometer (Berthold) and normalized to the amount of Renilla Luciferase Activities as described [44]. The results were representative of three independent experiments, each in triplicates.

### Fusion protein expression, polyclonal anti-DreI antiserum preparation and Western blotting

The plasmid pET-32a(+)-*DreI* was transformed into DE3 (BL21) *E. coli* strain. The recombinant fusion protein *DreI*-His was induced by isopropyl-b-D-thiogalactopyranoside (IPTG) and purified by Ni^2+^-NTA affinity chromatography (Novagen). The purified fusion protein was applied to immunized white rabbit to produce polyclonal anti-DreI antiserum according to a previous report [20].

For Western blotting, equal amounts of protein extracts from ZFL cells stimulated with poly I:C or not were separated by 12% SDS-PAGE gels and then transferred to a polyvinylidene difluoride (PVDF) membrane (Millipore). The PVDF membrane was blocked in freshly prepared TBST buffer containing 5% nonfat milk for 1 h at room temperature, incubated with primary antibody at 4°C overnight. After three times wash with TBST buffer, the membrane was incubated with secondary antibody for 1 h at room temperature. Finally, the membrane was stained with ECL system after another three washes with TBST buffer. For specificity detection, the membrane was incubated with anti-DreI antiserum that had been preabsorbed with excess antigen (purified DreI-His) at 4°C overnight.

### Immunofluorescent microscopy

ZFL cells were seeded overnight on microscopic coverglass in 6-well plates, and transfected with poly I:C, or infected with SVCV or RGV. Forty-eight hours later, cell monolayers were fixed with 4% paraformaldehyde (PFA) for 15 min, treated with 0.2% Triton X-100 for 15 min, blocked with 10% normal goat serum for 1 hour and incubated with polyclonal anti-DreI rabbit serum overnight at 4°C. Then, the cells were incubated with flourescein-labeled goat anti-rabbit IgG for 2 h in dark at room temperature. After three 10-min washes with PBS, the cells were stained with Hochest 33342 (Sigma) for 10 min in dark. Finally, the cells were examined under a Leica DM IRB fluorescence microscope.
